# Development of *Sparganothis sulfureana* (Lepidoptera: Tortricidae) on Cranberry Cultivars

**DOI:** 10.3390/insects9010004

**Published:** 2018-01-02

**Authors:** Erin E. McMahan, Christelle Guédot

**Affiliations:** Department of Entomology, University of Wisconsin-Madison, 1630 Linden Drive, Madison, WI 53706, USA; emcmahan@gmail.com

**Keywords:** Antibiosis, Sparganothis fruitworm, *Vaccinium macrocarpon*, host plant resistance

## Abstract

Sparganothis fruitworm (*Sparganothis sulfureana* Clemens) (Lepidoptera: Tortricidae) is a serious pest of cranberry (*Vaccinium macrocarpon* Aiton), a native North American fruit cultivated in northern regions of the United States and southeastern Canada. This study assessed antibiosis in several cranberry cultivars commonly grown in Wisconsin. Five cultivars previously shown to host different levels of populations of *S. sulfureana* in commercial cranberry were assessed in this study to evaluate the performance of *S. sulfureana* amongst these cultivars. We measured growth and time to developmental stages of newly emerged larvae to adulthood on selected cranberry cultivars in the laboratory. There was no difference in the rates of survival to pupation and to adult emergence among any of the cultivars tested. Mid-instar larvae that fed on the cultivar ‘Ben Lear’ were heavier than those feeding on ‘GH-1’, ‘Stevens’, or ‘HyRed’, and larvae that fed on ‘Mullica Queen’ were heavier than those feeding on ‘HyRed’. However, there were no significant differences in pupal weights or in the number of days from neonate to adult emergence among varieties. Therefore, this study did not provide evidence of antibiosis among the cultivars tested, and found that larval weight was not correlated with other measurements of performance.

## 1. Introduction

An essential part of Integrated Pest Management (IPM) is the strategy of host plant resistance (HPR), by which growers plant crop cultivars with heritable properties that protect them against damage from insects, fungi, and pathogens [1]. Resistant cultivars of many crops worldwide have been used to reduce damaging insect populations [2]. One mechanism of HPR is antibiosis, by which physical or chemical properties of a plant can kill or adversely affect the development of an insect [1]. Laboratory assays measuring the growth rate and survival of insects on different plant cultivars are often used to determine whether antibiosis is responsible for resistance. These assays have been used to find cultivars of cotton [3], corn [4,5], and numerous other crops that exhibit resistance towards insect pests.

The American cranberry (Ericaceae: *Vaccinium macrocarpon* Aiton) is a native North American fruit, which has only been cultivated from wild populations for about 200 years [6,7]. Cranberry is a wetland plant, and its cultivation depends on flooding several times per year, so commercial marshes are often embedded in natural wetlands that can easily be disrupted by pesticides and fertilizers [8]. Despite the importance of HPR in other crops and the growing interest in alternative pest management strategies from cranberry growers, fewer than 20% of cranberry growers use resistant cultivars as a pest management strategy for diseases [9], and little research has focused on addressing pest problems through HPR. In the early 1900s, false blossom disease, transmitted by the blunt-nosed leafhopper *Limotettix (=Scleroracus) vaccinii* (Van Duzee) (Hemiptera: Cicadellidae), became a major threat to the cranberry industry. This led to the establishment in 1929 of the first USDA cranberry breeding program with the purpose of searching for traits that provided resistance to the insect vector [10]. Laboratory bioassays found differences in resistance among several different native varieties [11], which were used to breed new resistant cultivars. Since that original program, the focus of breeding has shifted from insect resistance to traits like high productivity, fruit quality, and color [10].

Recent studies have shown differences in resistance among cranberry cultivars grown on the East Coast to gypsy moth (*Lymantria dispar* Linnaeus) (Lepidoptera: Eribidae), a damaging East Coast pest, and have suggested a possible chemical basis for resistance [12,13]. Rodriguez-Saona et al. (2011) [13] measured the phytochemical composition of the foliage of several different cranberry cultivars, and reared gypsy moth larvae for seven days on these cultivars. In their study, the insects gained the most weight feeding on the newest hybrid cultivar, ‘Crimson Queen’, suggesting that resistance traits may have been lost in breeding for other qualities. However, larvae also gained the most mass feeding on its native parent, ‘Ben Lear’, suggesting that susceptibility may be inherited. Concentrations of phenolics, plant hormones, and volatiles differed among the different cultivars, but there was not a significant correlation between levels of these compounds and insect performance. Neto et al. (2010) [12] found higher levels of several phenolic compounds in the cultivar ‘Early Black’ than in ‘Howes’, and observed significantly less gypsy moth feeding damage on ‘Early Black’. These studies demonstrate the unclear relationship between concentrations of phytochemicals and insect resistance in cranberry, and indicate a need for more research in cranberry HPR.

*Sparganothis sulfureana* (Clemens) (Lepidoptera: Tortricidae), commonly known as sparganothis fruitworm, is a serious pest of commercial cranberry, alongside *Acrobasis vaccinii* Riley (Lepidoptera: Pyralidae), also known as cranberry fruitworm. *Sparganothis sulfureana* is a native North American species, and is highly polyphagous, with host plants including cranberry and blueberry (Ericaceae), apple (Rosaceae), alfalfa (Fabaceae), celery (Apiaceae), and pine (Pinaceae), among others [14]. Studies suggest, however, that it prefers to feed on cranberry, blueberry, and weeds such as loosestrife (Lythraceae) within cranberry beds [15]. *Sparganothis sulfureana* is a bivoltine pest that overwinters as a first instar larva and feeds on new foliar growth in the spring, reducing the photosynthetic capacity of the plants. The second, more damaging larval generation burrows into berries as soon as the fruit begins to enlarge. Each larva of this generation hollows out and destroys 3–5 berries during its development, causing significant economic damage to growers [14]. Rarely, a third generation may occur later in the fall [16].

To control this damaging pest, many cranberry growers use IPM strategies such as the careful monitoring of insect populations [9] and flooding cranberry beds in the spring [17,18]. Pheromone-based mating disruption is another strategy currently under development [19,20]. Chemical control is the most commonly utilized method for management, and to combat more serious infestations, growers often rely on several in-season sprays of broad-spectrum organophosphate insecticides such as chlorpyrifos, diazinon, and acephate [21,22]. In parts of the East Coast, the increasing resistance of *S. sulfureana* to organophosphate insecticides has decreased the effectiveness of these chemicals [23]. The use of these broad-spectrum insecticides also harms beneficial insects, including pollinators and the natural enemies that can contribute significantly to *S. sulfureana* control [16,24]. The use of insecticides can also have adverse environmental and human health impacts, and can limit cranberry sales to foreign markets if pesticide residues surpass the Maximum Residue Limits (MRLs) enforced by these markets [19]. Given the many problems for growers associated with organophosphate insecticides, it is important to continue to develop novel IPM strategies including HPR.

The state of Wisconsin produces ca. 60% of the nation’s cranberries [8] and there are over 140 native and cultivated cranberry varieties [7]. ‘Stevens’ is currently the most widely grown cultivar in the cranberry industry and a product of the original breeding program. The native variety ‘Ben Lear’ is still commonly grown and demonstrated more susceptibility than ‘Stevens’ to gypsy moth feeding [13]. ‘GH-1’ is a recent cultivar released by a Wisconsin cranberry grower and breeder [25], and ‘HyRed’ and ‘Mullica Queen’ are both recently released cultivars from the University of Wisconsin-Madison and Rutgers University, respectively [26,27]. These newer cultivars are growing in popularity amongst growers and it has been suggested that varieties bred recently for yield, size, and color may have lost insect resistance traits [13]. Because cranberry growers routinely remove lower performing native varieties for newly-bred cultivars, there is a need to assess cultivar susceptibility to commercially important pests.

Using pheromone traps in commercial cranberry marshes, higher population densities of adult male *S. sulfureana* were reported in beds of ‘Stevens’ and ‘GH-1’ than in beds of ‘Ben Lear’, ‘Mullica Queen’, or ‘HyRed’, indicating higher overall *S. sulfureana* populations in these cultivars [28]. It is unclear whether these population differences are due to resistance in these cultivars, and more research is necessary to determine what resistance mechanisms may be at work, e.g., phenological resistance, antibiosis, or antixenosis.

The goal of this research was to assess *S. sulfureana* growth and time to development in the laboratory on five of the most commonly grown cultivars in the leading cranberry-producing region, i.e., ‘Stevens’, ‘Ben Lear’, ‘GH-1’, ‘HyRed’, and ‘Mullica Queen’. Based on field observations [28], we hypothesized that our laboratory tests would demonstrate better insect performance on the cultivars ‘Stevens’ and ‘GH-1’, which showed higher adult moth populations in commercial marshes. This would help demonstrate whether or not these population differences can be attributed to antibiosis in some cultivars.

## 2. Methods and Materials

### 2.1. Plants

Five cranberry cultivars commonly grown in Wisconsin—‘Stevens’, ‘Ben Lear’, ‘GH-1’, ‘HyRed’, and ‘Mullica Queen’—were assessed for resistance. Due to its prevalence in production and its use as a standard cultivar in other research [13,27,29], the cultivar ‘Stevens’ served as the control in this study. All plants used in the experiment were rooted at the same time.

Rooted cuttings of all five cultivars were acquired from a commercial nursery (Evergreen Nursery Co. Inc., Sturgeon Bay, WI, USA) in the spring of 2015. Cuttings all came from the propagator’s stock, with the exception of the ‘Ben Lear’ cuttings, which were collected by the propagator from a commercial marsh the previous fall, and were overwintered and planted under the same environmental conditions as the other cultivars. Cuttings were then transplanted into 10 cm × 36 cm × 51 cm black plastic flats (Dyna-flat™, Kadon Corp., Dayton, OH, USA) in a medium of 80% peat moss, 10% commercial substrate (Metro-Mix^®^ 366 P Series; Sun Gro^®^, Agawam, MA, USA), and 10% autoclaved sand collected from a commercial cranberry marsh. Plants were grown in a controlled environment greenhouse (22–25 °C, 16:8 Light: Dark photoperiod), watered at least every other day, and fertilized weekly, or as needed with Miracid fertilizer (Scott’s Miracle Gro., Marysville, OH, USA).

### 2.2. Insects

The source of the *S. sulfureana* larvae used in the experiment was a colony that was started with larvae collected from a cranberry marsh near Warrens, Wisconsin in the summer of 2013, and kept in a Percival I-36LLVLC8 growth chamber (Percival Scientific Inc., Perry, IA, USA) with a 16:8 L:D photoperiod at 24 °C. Insects were reared on a wheat germ-based diet (Stonefly Heliothis Diet, Ward’s Science, Henrietta, NY, USA) in 29.6-mL lidded clear plastic cups (Dixie, Atlanta, GA, USA) to eliminate preference for any cranberry cultivar. Pupae were removed and allowed to emerge in mesh-lidded 355-mL clear plastic cups (Solo Cup, Lake Forest, IL, USA). Upon emergence, moths were allowed to mate and oviposit, and eggs were checked daily for hatch. Immediately after hatching, neonates were used in the experiment.

### 2.3. Hydroponic System

**Figure 1:**
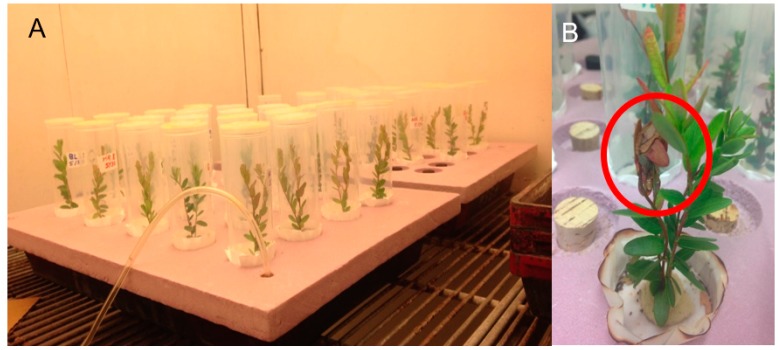
(**A**) Experimental hydroponic system setup. Pink foam boards are set on top of plastic flats filled with deionized water. The aeration tube leads to an aquarium pump (not shown); (**B**) Individual replicate with the vial removed. A webbed upright, which late instar larvae build to feed concealed, is visible in the picture within the circle.

The experiment employed a hydroponic growing system made with 2.54 cm-thick stiff foam insulation (Owens Corning, Toledo, OH, USA) with 20 or 35 circular indentations (4.6 cm in diameter), each with a hole (1.8 cm in diameter) through the center (Figure 1a). Foam was grooved to fit snugly on the top of a black plastic flat with dimensions of 6.5 cm × 36 cm × 51 cm (Dyna-flat™, Kadon Corp., Dayton, OH, USA) containing 8.9 L of deionized water. Water was aerated using an aquarium pump (Elite 801 Air Pump, Rolf C. Hagen Corp., Mansfield, MA, USA) to provide oxygen to the plants’ roots. A circle of filter paper 9 cm in diameter (Qualitative Circles, rating 1, Whatman, Little Chalfont, Buckinghamshire, UK) with a 1.8 cm hole in the center was pressed into the bottom of each indentation to allow for easy visualization of insect frass, which is indicative of larval feeding. For each replicate, two clipped cranberry uprights of the same cultivar were inserted into each hole to provide access to water below, with stems wrapped in foam (Future Foam, Council Bluffs, IA, USA) that expanded to provide a snug fit and seal the hole. At least 7 cm of stem were submerged in the water. Before adding uprights to the experiment, they were rinsed thoroughly in deionized water, dried, and agitated to remove any potential debris or insects such as thrips. Each replicate of two uprights was enclosed in an individual rearing container which consisted of a 221.8-mL clear plastic vial (Squeezetops Pharmacy Vials, United States Plastic Corp., Lima, OH, USA) with the base removed and the top removed and covered with thrips mesh (GreenTek, Janesville, WI, USA) to allow for ventilation. Six of these hydroponic systems were set up in a growth chamber (22–24 °C, 16:8 L:D, 27–67% Relative Humidity).

### 2.4. Developmental Rate Assay

Twenty replicates (vials) of each of the five cultivars were set up in the hydroponic system. One neonate *S. sulfureana* larva was added to each vial using a paintbrush. Neonates did not all hatch on the same day, so an equal number of replicates for each cultivar were added on 30–31 May 2014. Larvae were allowed to feed freely, and an effort was made to minimize disturbance. Larvae were checked daily to assess survival, and fresh uprights were added every three days or as needed to maintain optimal food quality. No more than four uprights accumulated before the old ones were removed. An exception was made if the larva had woven several uprights together (Figure 1b), and uprights could not be removed without significant larval disturbance.

After 16 days, midway through their development, larvae were weighed, and returned to the uprights to continue feeding. Upon pupation, insects were removed from the uprights, and pupae were weighed, sexed [30], placed in individual petri dishes (35 × 15 mm), and returned to the growth chamber. Petri dishes were checked daily for adult emergence, upon which total days to emergence and survival to adulthood was measured. To avoid delaying pupation by disturbing larvae inside their leaf tents, final instar larvae were not observed for pupation every day. Instead, if more than three days had passed without evidence of feeding (indicated by the presence of frass or new leaf damage), tents were carefully examined to look for pupae. These precautions did not allow for a recording of the exact number of days to pupation, so the number of days from setup to adult emergence was used as an indicator of developmental time. The ratio of male to female pupae in each cultivar was recorded, although larvae were initially distributed randomly.

### 2.5. Statistical Analysis

Differences in insect survival rates among cultivars were analyzed using a Chi-squared test (PROC LOGISTIC; Version 9.4, SAS Institute Inc., Cary, NC, USA) [31]. Larval weight, pupal weight, and days to emergence were averaged for each cultivar, square root transformed, and analyzed with an analysis of variance (ANOVA) using PROC MIXED (Version 9.4, SAS Institute Inc. 2014). If significant *p* values were found, Fisher’s LSD (Least Significant Difference) was used to further analyze differences between cultivars [32].

## 3. Results

### 3.1. Survival Rates

**Figure 2:**
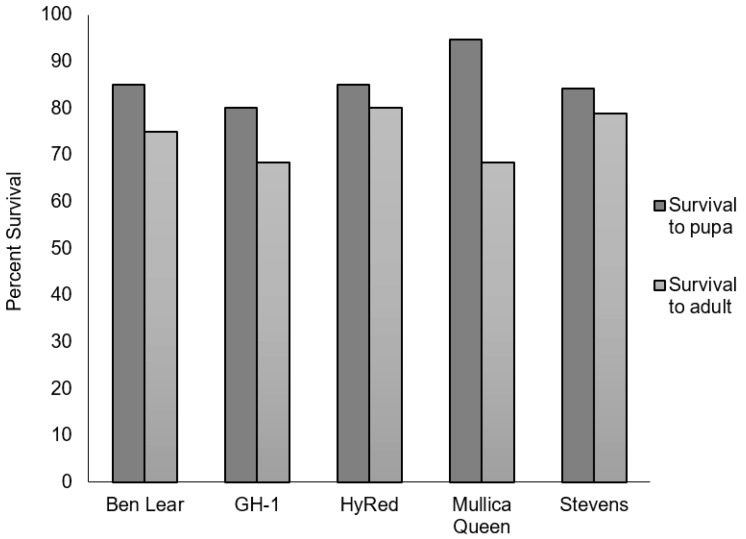
Percent of larvae that survived to pupation and to adult emergence for each cultivar. Dark gray bars indicate survival to pupation while light gray bars indicate survival to adult emergence.

Percent survival from neonate to pupation (not including those inadvertently killed by researchers) ranged from 80% to 94.7% among cultivars with an average survival rate of 85.8%. There was no significant difference in percent survival to pupation among cultivars (*Χ*^2^ (*df* = 4) = 2.14, *p* = 0.71) (Figure 2). Survival from neonate to adult emergence ranged from 68.4–80% among cultivars, with an average of a 74.2% survival rate. ‘Mullica Queen’ had the lowest adult emergence and many pupae that fed on this cultivar were deformed; however, there was no significant difference in survival among cultivars (*Χ*^2^ (*df* = 4) = 1.13, *p* = 0.89) (Figure 2).

### 3.2. Growth and Developmental Time

**Figure 3:**
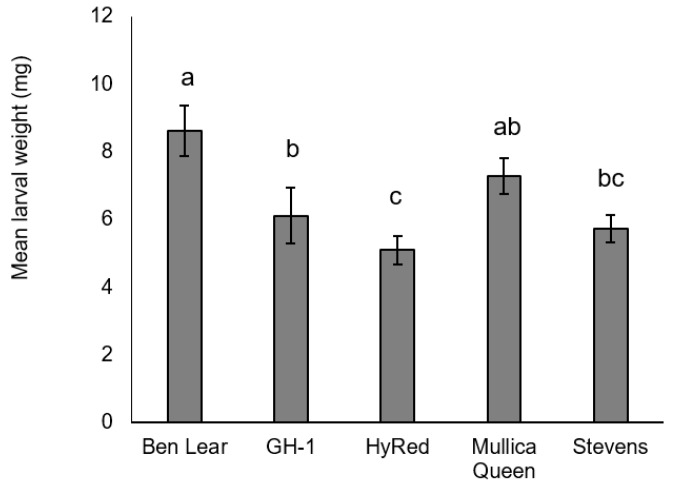
Mean larval weight (± SEM) at 16 days of *S. sulfureana* that fed on selected cranberry cultivars. Different letters indicate significant differences among cultivars (*F*_4,68_ = 5.42, *p* = 0.0008). For each cultivar, *n* = 18–19.

There was a significant difference between cultivars in larval weights at 16 days (*F*_4,68_ = 5.42, *p* = 0.0008) (Figure 3). Larvae feeding on ‘Ben Lear’ were significantly heavier (mean ± SEM; 8.62 ± 0.75 mg; *n* = 19) than those feeding on ‘GH-1’ (6.11 ± 0.83 mg; *n* =18), ‘Stevens’ (5.73 ± 0.4 mg; *n* = 18), or ‘HyRed’ (5.10 ± 0.41 mg; *n* = 18). Larvae feeding on ‘Mullica Queen’ were significantly heavier on average (7.29 ± 0.53 mg; *n* = 18) than those feeding on ‘HyRed’.

**Figure 4:**
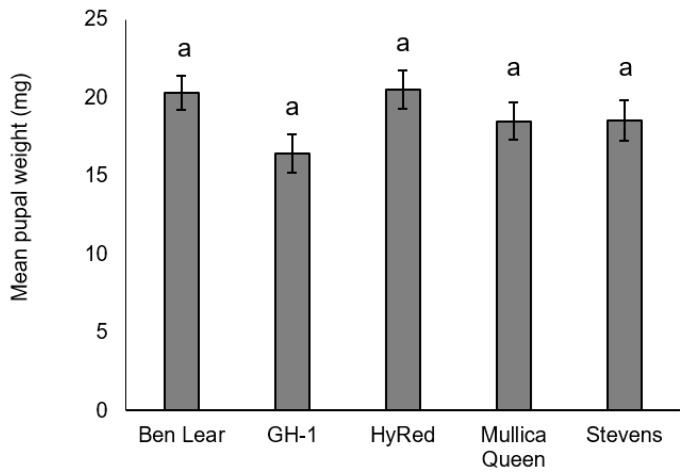
Mean pupal weight (± SEM) of *S. sulfureana* that fed on different cultivars. Different letters indicate significant differences among cultivars (*F*_4,60_ = 1.83, *p* = 0.13). For each cultivar, *n* = 18–19.

**Figure 5:**
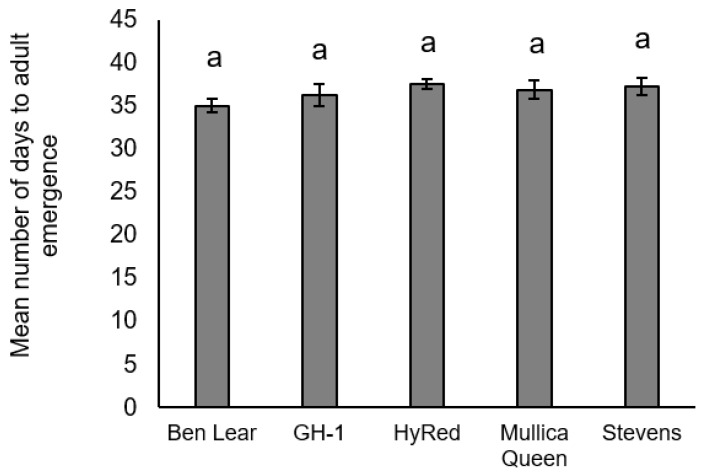
Mean (± SEM) days from neonate to adult emergence of *S. sulfureana* that fed on different cranberry cultivars. Different letters indicate significant differences among cultivars (*F*_4,48_ = 1.62, *p* = 0.19). For each cultivar, *n* = 17–19.

There was no significant difference in pupal weight among cultivars (*F*_4,60_ = 1.83, *p* = 0.13) (Figure 4). There was, however, a non-significant lower pupal weight in ‘GH-1’ compared to the other cultivars. There was no significant difference in average days to adult emergence among cultivars (*F*_4,48_ = 1.62, *p* = 0.19) (Figure 5).

## 4. Discussion

Overall, our results did not demonstrate differences in resistance through antibiosis among the selected cranberry cultivars towards *S. sulfureana*, an important insect pest of cranberry, despite differences observed in field populations of this pest among the same varieties [28]. We provide the first evidence that among the major cranberry cultivars grown in the state of Wisconsin, there are no significant differences in *S. sulfureana* performance in a laboratory setting. Interestingly, although *S. sulfureana* mid-instar larval weights differed among cultivars, the trends were not supported throughout the development of the insects. By the time the insects pupated, there was no significant difference among any of the cultivars tested.

We found that larvae feeding on ‘Ben Lear’ were heavier than those feeding on other cultivars, which supports previous results obtained with gypsy moth [13]. The previous study also found that larvae were heavier after feeding on the cultivars NJS98-23 (a recent hybrid since released as ‘Crimson Queen’) and ‘Ben Lear’ than on other cultivars, and suggested that the susceptibility of ‘Ben Lear’ was inherited by its offspring, ‘Crimson Queen’. The recent cultivar ‘HyRed’ also has ‘Ben Lear’ and ‘Stevens’ as parents, yet in our study, larvae feeding on ‘HyRed’ weighed less than larvae on any other cultivar and weighed significantly less than larvae on ‘Ben Lear’. However, since any evidence of antibiosis was lost by pupation and adult emergence, ‘HyRed’ did not exhibit inheritance of resistance relative to the other cultivars.

This study reinforces the suggestion that short-term changes in larval weight may not accurately represent changes in the weight of the insect over its entire development, and that differences in larval weight gain alone may not be an accurate predictor of host plant resistance [33]. Farrar and Kennedy (1990) [33] found that only measuring larval weight after a set number of days might exaggerate the effect of a plant or plant chemistry on insect growth. Indeed, feeding on a diet containing alpha-tomatine caused a 47% weight reduction in *Heliothis zea* larvae after feeding for 10 days, but only a 4.6% reduction in pupal weight and a 15% increased time to development [33]. A study of the lappet moth on blueberry, another *Vaccinium* species, demonstrated a difference in larval weight gain and developmental time among cultivars, but found no significant differences in pupal weight among cultivars [34]. These findings suggest that measuring insect growth over the course of its entire development can more effectively assess antibiosis. To our knowledge, our study is the only one to carry out a bioassay of lepidopteran larvae in cranberry through adult emergence.

Several studies have demonstrated that early instar lepidopteran larvae are more sensitive to plant secondary compounds, including growth-inhibiting flavonoids and phenolics, in comparison to later instar larvae. These chemical defenses induced higher levels of mortality and decreased growth rates in these early instars [35,36,37]. However, Rodriguez-Saona et al. (2011) [13] found a difference in weight gain in early instar gypsy moth larvae among cranberry cultivars, but no correlation between caterpillar mass and total concentrations of phenolic acids or flavonols. Leaf toughness is also very important in deterring herbivory [38], and it has been suggested that leaf toughness could impact early instars more than later instars [39,40]. Subtle differences in plant chemistry and leaf toughness between cultivars may have been much more obvious in the mid-instar larvae, but the effect disappeared by the time the late instar larvae pupated. Leaf chemistry and toughness are known to change throughout the development of a plant [41] and these characteristics, along with cultivar characteristics, may affect the performance and development of insects that feed upon them. Indeed, another important Lepidopteran pest of cranberry, *Rhopobota naevana* Hübner, prefers to feed on younger cranberry leaves and their development and survival are both affected by the age of the leaves, with a slower developmental time and higher mortality reported with older cranberry leaves [42]. 

At the initiation of our experiment, neonates were randomly allocated to each experimental container without being sexed, thus we had different sex ratios on each cultivar. In other lepidopteran species, females are generally larger than males [43,44] and indeed, in our experiment, *S. sulfureana* female pupae were significantly larger than the males (*t*_80_ = 6.70, *p* < 0.0001), possibly impacting average larval and pupal weights. However, in this study, the only significant difference among cultivars was in the weight of larvae at 16 days, and no difference was found between the weights of male and female larvae at that stage (*t*_80_ = 0.21, *p* = 0.84). When the sexes were separated and analyzed as pupae, there was no significant difference in weight among cultivars for either males (*F*_4,41_ = 0.16, *p* = 0.96) or females (*F*_4,31_ = 1.64, *p* = 0.19), and the lack of significant difference with sexes pooled was confirmed.

This laboratory experiment measured larval performance of *S. sulfureana* on the foliage only and did not include performance on the fruit of the cranberry plant. Thus, this study reflects the activity of the first generation of insects in the field that feed exclusively on the foliage before the cranberries form. The success of the first generation will likely impact the population size of the second, more damaging, generation. Pupal size is positively correlated with adult female fecundity in many insect species [45,46], thus, the pupal weight of the first generation may have an impact on the number of eggs and therefore the population size of the second generation. A longer time to development can increase the amount of time the insect is in a vulnerable larval stage, expanding the window for management and exposure to the natural enemies that can reduce pest populations. Since our results showed no difference in pupal weights or time to development among the selected cultivars, this suggests the antibiosis in the cultivars tested herein will likely not have an impact on the population of the second generation, though, as discussed above, changes in plant chemistry and physical characteristics may change over time and affect insect response.

A recent field study found lower *S. sulfureana* adult male population densities in beds of ‘Ben Lear’, ‘Mullica Queen’, and ‘HyRed’ compared to beds of ‘Stevens’ and ‘GH-1’ [28]. The results presented herein do not support these field observations in the same cultivars. However, there is no direct link between field adult populations and larval developmental rate in controlled laboratory settings. Indeed, larvae in laboratory feeding trials were provided with new and healthy foliage in a similar stage of growth for all cultivars; however, in the field, the cultivars exhibit minor differences in phenology, with cultivars such as ‘Ben Lear’, ‘HyRed’, and ‘Mullica Queen’ bearing fruit slightly earlier than other cultivars. A number of components besides antibiosis, including plant and insect phenology, natural enemy populations, bed age, or history of outbreaks, could be responsible for different field populations among cultivars [28]. 

This study did not observe antibiosis in any of the five commonly grown cranberry cultivars tested. However, there are over 140 known cultivars of cranberry, a majority of them selected from the wild [7], so many more can be assessed for resistance. Cranberry is unique in that there are still native varieties growing in natural landscapes, which may offer inherent sources of resistance. The cultivars in this study were measured for resistance against one of the most economically important pest insects in cranberry, *S. sulfureana*, yet there are several other important pest species, including cranberry fruitworm (*Acrobasis vaccinii* Riley) and blackheaded fireworm (*R. naevana*), that have yet to be tested.

## 5. Conclusions

In this study, we did not find evidence of antibiosis in *S. sulfureana* to the selected cranberry cultivars evaluated. An unforeseen outbreak such as that of false blossom disease has the capacity to threaten the entire cranberry industry, and host plant resistance has rescued growers in the past [10]. Genetic modification has thus far not been shown to be a viable option in cranberry [47,48]. Therefore, it is imperative to continue the search for resistance characteristics in different domestic and wild cranberry cultivars. With the desire of growers to be more sustainable and reduce pesticide usage, research into host plant resistance and other IPM tools in cranberry remains essential.
